# Direct Separation of Pregabalin Enantiomers Using a Zwitterionic Chiral Selector by High Performance Liquid Chromatography Coupled to Mass Spectrometry and Ultraviolet Detection

**DOI:** 10.3390/molecules21111578

**Published:** 2016-11-19

**Authors:** Lakshmi Narayana Chennuru, Thirupathi Choppari, Ramakrishna Prasad Nandula, Tong Zhang, Pilar Franco

**Affiliations:** 1 Daicel Chiral Technologies India, Pvt Ltd, Lab n° 4A, IKP Knowledge Park, Shameerpet, Hyderabad 500078, India; lakshminarayana@chiral.daicel.com (L.N.C.); thirupathi@chiral.daicel.com (T.C.); ramakrishna@chiral.daicel.com (R.P.N.).; 2 Chiral Technologies Europe, Parc d’Innovation, Bd. Gonthier d’Andernach, Illkirch F‑67400, France; tzhang@chiral.fr

**Keywords:** pregabalin, zwitterionic selector, chiral stationary phases, direct enantiomer separation by HPLC, mass spectrometry, ultraviolet detection, method optimization

## Abstract

The chromatographic resolution of pregabalin enantiomers has been often achieved by derivatization of the molecule, in order to reach enough sensitivity at low concentrations of the minor enantiomer present in the active principle. In the present article, the development and optimization of two liquid chromatographic methods are presented for the direct resolution of pregabalin enantiomers on a chiral stationary phase (CSP) containing a zwitterionic selector derived from cinchona alkaloid and sulfonic acid (CHIRALPAK ZWIX). The key parameters for the separation as well as the compatibility of chromatographic conditions with different detection modes (ultraviolet and mass spectrometry) were investigated. The resulting methods were found to be selective, of high performance and low limits of detection (2 µg/mL by UV and 1 ng/mL by MS, respectively) and quantification (6 µg/mL by UV and 5 ng/mL by MS, respectively) for the minor enantiomer which is considered as a chiral impurity.

## 1. Introduction

Pregabalin (PRG), (*S*)-3-(aminomethyl)-5-methylhexanoic acid), is an antiepileptic and analgesic drug. It is a structural analogue of the endogenous inhibitory neurotransmitter γ-aminobutyric acid (GABA), which is involved in the regulation of brain neuronal activity [1]. Under the trade name Lyrica^®^, this drug was approved by Food and Drug Administration (FDA) in 2007 for the treatment of spinal cord injury and central nervous system (CNS) disorders as well as for the treatment of neuropathic pain, among many other therapeutic activities associated with physiological conditions and psychomotor stimulants [1,2,3,4].

**Figure 1:**
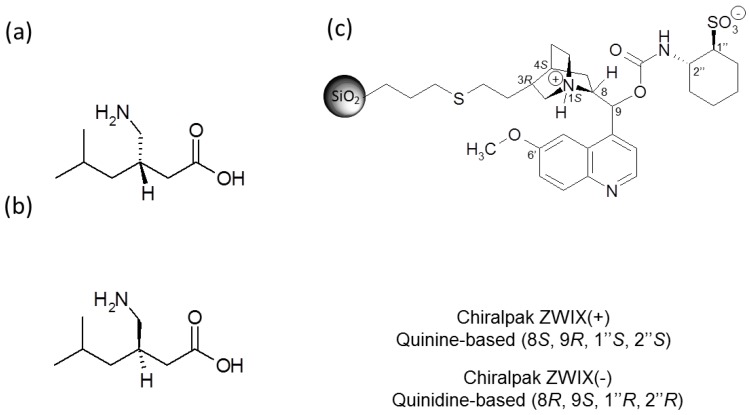
Chemical structures of PRG (**a**), its (*R*)-enantiomer (**b**), and the chiral selectors in ZWIX columns (**c**).

Enantiomers of a drug are often known to have differences in pharmacological actions, in pharmacokinetics, in toxicities and in metabolism. Therefore, the use of a single isomer of a given drug is usually recommended for clinical use [5]. PRG is the (*S*)‑enantiomer of a γ-amino acid containing one stereogenic carbon (Figure 1a). While PRG is proved to have significant clinical relevance, its (*R*)-enantiomer (Figure 1b) has been reported to be 10 times less active in biological assays [6]. The (*R*)-enantiomer is therefore considered as an impurity issuing from PRG synthesis and should be subjected to strict drug quality control via effective analytical methods for its quantification.

The major challenges in analytical method development to separate PRG from its (*R*)-enantiomer are tightly related to its amphoteric nature as well as the lack of a strong chromophore in its structure for sensitive UV detection. Several achiral analytical separation methods have been developed for the purpose [7,8,9], all employing the derivatization approach. Different derivatization agents were examined to enhance the detection properties or sensitivities and the separation of PRG from the impurities. For instance, picryl sulfonic acid (PSA) was used as the derivatization agent for the separation of PRG in serum samples by HPLC on a C8 column [7]. Vermeji et al. reported the derivatization method by *o*-phtaldialdehyde (OPA) and 3-mercaptopropionic acid for simultaneous determination of PRG, gabapentin and vigabatrin in human serum via HPLC‑fluorescence detection and the application of these methods to pharmacokinetic study [8]. Another fluorescence detection method was developed for the separation of PRG and its impurities upon their derivatization with *o*-phtaldialdehyde/2-mercaptoethanol [1]. A cyclodextrin-modified capillary zone electrophoresis (CZE) method was developed to separate PRG enantiomers upon their derivatization with tosyl and dansyl chloride [6]. Several mass spectrometric methods coupled with gas chromatography (GC-MS) and liquid chromatography (LC-MS) were also demonstrated to investigate PRG and the related substances in human plasma [2,9]. An additional example to be mentioned is the diastereoisomer formation of PRG enantiomers by reacting with Marfey’s reagent. In this latter case, the resulting diastereoisomers are separated by using a C18 column for the quality control of PRG [10,11].

A more attractive and straightforward approach would be the direct enantiomeric separation or determination of PRG enantiomers by chromatography. However, much fewer reports could be found in this regard. A direct enantiomeric separation of PRG was achieved with good detection sensitivity (50 ng/mL) by using a macrocyclic glycopeptide chiral stationary phase (CSP) in HPLC/MS/MS, but the narrow resolution degree (R_s_ = 1.2) led to incomplete separation between the two enantiomers [12].

With the introduction of zwitterionic chiral selectors derived from cinchona alkaloids and sulfonic acids and supported on silica (CHIRALPAK ZWIX, Figure 1c), we undertook an investigation of the potential direct resolution of PRG enantiomers by HPLC. As described in the literature, such CSPs exhibit remarkable stereoselectivity for amphoteric molecules, especially amino acids and peptides [13,14,15,16]. The successful resolution of PRG enantiomers on ZWIX columns was presented in 2013 [14] and subsequently reported in a research paper published in early 2014 [15].

These conditions were used as a starting point to develop a direct, improved and effective method for resolution of the PRG enantiomers with two main goals:

(1) Optimization of HPLC conditions to get the method compatible with MS detection.

(2) Identification of an efficient HPLC method compatible for UV detection with enhanced sensitivity that could be proposed to laboratories not equipped with mass spectrometry detectors.

Extensive method optimization was carried out in our laboratories in India in 2013. Promising results were obtained and communicated to a number of potential users after checking the main chromatographic parameters. In late 2014, one research team in India [17] published the validation of a UV method fitting the chromatographic conditions we communicated (no comment was made about the method’s development in such conditions). The objective of this current report is to disclose the main lines of the method development, the critical parameters in the two mobile phase systems (one compatible with UV and the other with MS detection) and the final conditions that can be successfully applied.

## 2. Results and Discussion

As part of our investigation on the separation of PRG enantiomers, several different chiral columns had been experimented with, including polysaccharide-derived CSPs, crown ether–based CSPs, as well as the cinchona‑based CSPs. Previous investigations on certain polysaccharide-derived columns did not achieve a baseline resolution of PRG enantiomers [10]. A recent screening study on the immobilized amylose- and cellulose-derived CSPs was leading to a successful separation of PRG from its (*R)*-enantiomer on a CHIRALPAK IE column (amylose tris(3,5‑dichlorophenylcarbamate) immobilized on spherical silica, using a *n*-hexane/EtOH/TFA/NH_3_ (80/20/0.3/0.3, *v*/*v*/*v*/*v*) mobile phase. However, the chiral impurity was eluted after the main PRG peak. The method’s sensitivity was found to be poor in such an elution profile with UV detection due to the lack of strong chromophores in the analyte and thus did not allow quantification below a 1% level of (*R)*-enantiomer. A nice separation was obtained on a CROWNPAK CR-I(+) column with the right elution order (*R)*-enantiomer eluted in front of PRG) but with the inconvenience of the reduced recovery of the analytes, probably caused by the product’s degradation under the strong acidic mobile phase conditions of aqueous perchloric acid (pH 1)/acetonitrile (80/20, *v*/*v*).

In the case of CHIRALPAK ZWIX columns, baseline resolution was achieved, with a reversed elution order observed between the ZWIX(+) and ZWIX(−) columns which contain, respectively, the zwitterionic chiral selectors behaving as pseudo-enantiomers [13,15]. Owing to the favorable elution order (minority (*R)*-isomer eluted first) found on the CHIRALPAK ZWIX(+) column, such a column was chosen for further investigation.

In good accordance with the description in the literature [13,15,16], methanol was found to be an essential component in the mobile phases on ZWIX columns. The addition of low percentages of water was beneficial for modulating retention, enhancing solubility of the analyte in the mobile phase and developing an LC-MS–compatible method. Due to the intramolecular counter-ion effect of the chiral selectors, use of acidic and basic additives, such as formic acid and ammonium formate or diethylamine, is recommended to modulate the ion pairing and ion exchange mechanisms between the selector and the analyte molecules. In addition to the mobile phase composition, it was necessary to consider the detection viability and sensibility of the separation method. The method optimization of these aspects is actually the topic of the current publication.

### 2.1. LC-MS–Compatible Method

Based on the reported separation conditions of PRG enantiomers on the ZWIX(+) column [15], the method optimization was carried out for enhancing MS detection sensitivity by considering several chromatographic parameters, mainly including the additives, the temperature and the water content in the MeOH-based mobile phase.

The combination of formic acid and ammonium formate represents a convenient option for proper ionization and MS detection of the target compounds. Using MeOH/H_2_O (98/2, *v*/*v*) as the bulk mobile phase, the concentration of these mobile phase additives was varied, in equal molar concentration from 5 mM to 25 mM with increments of 5 mM. The effects induced by the additive concentration on retention times (t), on the resolution degree (R_s_) of the enantiomers, as well as on the signal-to-noise (S/N) ratio at UV 210 nm are summarized in the first section of Table 1. The enhanced values in R_s_ and in S/N were obtained by decreasing the additive concentration. The mobile phase containing 5 mM of formic acid and ammonium formate was chosen for further studies for the MS-compatible method, although the higher S/N values at 5 mM of additives is not essential for MS detection.

By keeping the identical bulk mobile phase of MeOH/H_2_O (98/2, *v*/*v*) containing 5 mM of each additive, the influence of temperature on the same parameters is shown in the second section of Table 1. Decreases in retention times, R_s_ and S/N were noticed with the increase in temperature. For the sake of convenience, 25 °C was selected as the column temperature for further studies.

Maintaining the 5 mM additive concentrations and a 25 °C column temperature, the effect of water in the mobile phase was examined. As expected, the increase in water content from 2% to 5% reduced the retention time and resolution but increased the S/N ratio (data in Section 3, Table 1). The method sensitivity was found to be good at 4% water rather than 5%, and hence MeOH/water 96/4 (*v*/*v*) was found to be the best choice for the bulk solvent composition.

**Figure 2:**
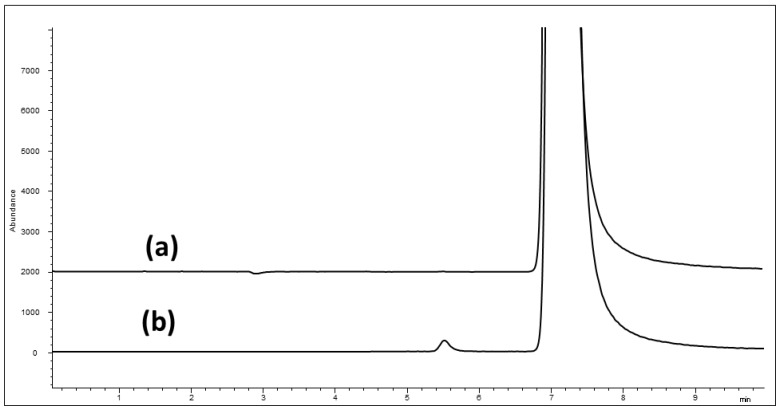
LC-MS chromatograms of: (**a**) PRG and (**b**) PRG spiked with 0.15% (*R*)-enantiomer. Conditions: (5 mM ammonium formate + 5 mM formic acid) in MeOH/water (96/4, *v*/*v*), 25 °C, CHIRALPAK ZWIX(+) (150 × 3.0 mm i.d.), MS detection on positive ion mode, SIM acquisition, *m*/*z* 160.

**Figure 3:**
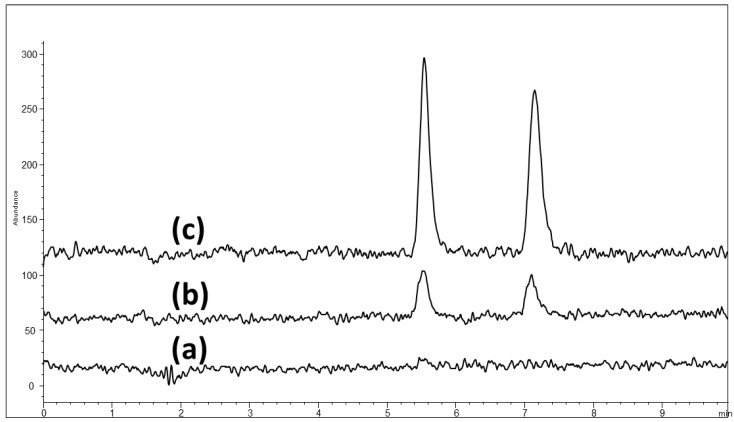
LC-MS chromatograms for LOD/LOQ determination with the racemic mixture: (**a**) blank chromatogram, (**b**) LOD chromatogram at 1 ng/mL, (**c**) LOQ chromatogram at 5 ng/mL. Chromatographic conditions are the same as in Figure 2.

As a summary, the optimized conditions compatible with MS detection were: 5 mM ammonium formate + 5 mM formic acid in MeOH/water (96/4, *v*/*v*) at a 25 °C column temperature on CHIRALPAK ZWIX(+) (150 × 3.0 mm i.d.). The separation of PRG enantiomers with MS detection can be represented in Figure 2b and Figure 3c, obtained by spiking 0.15% (*R)*-enantiomer into the PRG sample. Although the method validation is not the focus of the present work, the Limit of Detection (LOD) and Limit of Quantification (LOQ) were assessed to be, respectively, 1 ng/mL and 5 ng/mL with such an optimized LC-MS method.

It is worth noting that a resolution degree of 4.1 was achieved under these conditions, whereas the same parameter was limited to 1.2 in the previously described method on Chirobiotic T [12].

### 2.2. LC Method with UV Detection

The suitable MS-compatible method for PRG enantiomers without any derivatization was a positive step forward to guarantee the accurate quantification of the impurity. However, we understood that not all laboratories would be equipped with mass spectrometry. Therefore, the optimization of a simple HPLC method using UV detection to successfully quantify the chiral impurity of PRG was undertaken. As an outcome of the method optimization efforts, a UV transparent mobile phase based on 5 mM ammonium hydrogen orthophosphate could be proposed. The major challenge in using ammonium hydrogen orthophosphate would be its solubility in a methanol-rich mobile phase.

The bulk solvent composition used for MS detection (MeOH/water, 96/4, *v*/*v*) was not suitable for the easy and total dissolution of such an inorganic salt, even at very low concentrations. It was proposed to optimize the method by increasing the water in an appropriate proportion. A water percentage in the range of 5%–15% was investigated for this purpose and MeOH/water at 90/10 *v*/*v* was found to be the optimum by considering the salt solubility, the enantioselectivity and the very high eluting strength of water towards the analytes. As a consequence, a ZWIX column with larger dimensions (250 × 4 mm i.d.) was employed to ensure the good separation of PRG enantiomers with no change in the elution order. The effect of the temperature, salt concentration, injection volume and monitoring wavelength were also studied to achieve the optimum condition for the quantification of the chiral impurity using LC‑UV detection as it was performed in the previous case. In Table 2 the R_s_ and S/N results obtained by changing the sample concentration and the injection volume are summarized. The sample used was the Active Pharmaceutical Ingredient (API) or *(S)*‑PRG spiked with 0.15% of (*R)*‑enantiomer. The best results were found with the combination of the sample concentration of 10 mg/mL and the injection volume of 30 µL.

**Figure 4:**
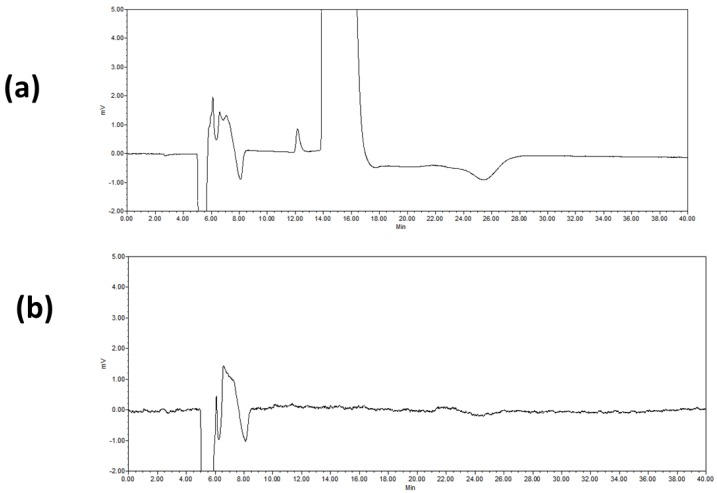
Chromatograms with optimized UV conditions: 5 mM ammonium hydrogen orthophosphate in MeOH/water (90/10, *v*/*v*), 10 °C, CHIRALPAK ZWIX(+) (250 × 4.0 mm i.d.), 0.5 mL/min, UV at 212 nm. (**a**) PRG spiked with 0.15% (*R*)-enantiomer, (**b**) blank chromatogram.

The optimized method for UV-detection can be summarized as follows: 5 mM ammonium hydrogen orthophosphate in MeOH/water (90/10, *v*/*v*) at a 10 °C column temperature on CHIRALPAK ZWIX(+) (250 × 4.0 mm i.d.) at 0.5 mL/min and 212 nm, with an injection volume of 30 µL of a solution prepared at 10 mg/mL of PRG dissolved in MeOH/water 50/50 (*v*/*v*) (Figure 4).

The LOQ of such a method was found to be 6 µg/mL. The method precision in terms of areas of the (*R)*-enantiomer was determined at the 0.15% level (six replicates). Integration results were consistent with the spiking amount. The % RSD of the (*R)*-enantiomer was found to be 2.1, indicating the excellent precision of the method.

As previously mentioned, the validation of the UV method under very similar conditions was published by another research group [17] and we will omit reporting the further verifications made in our hands with this original method. We have limited the description to the method development which was not reported before and include critical and non‑obvious optimization steps.

## 3. Materials and Methods

### 3.1. Chemicals

The mobile phases for liquid chromatography were prepared from HPLC grade solvents and additives. Methanol (MeOH), acetonitrile, *n*-hexane, ethanol, ammonium formate, ammonium dihydrogen orthophosphate, trifluoro acetic acid (TFA) and perchloric acid were purchased from Merck (Mumbai, India). Formic acid, ammonia (25% aq. ammonia solution, NH_3_) and ammonium formate were purchased from Rankem (Thane, India). Water was produced from Merck Millipore Milli Q water purification system (Merck Millipore, Molsheim, France). Pregabalin (PRG), PRG (*R)*-enantiomer and PRG racemate were gifted by Dr Reddy’s Laboratories Ltd, Hyderabad, India.

### 3.2. Instrumentation and Chromatographic Conditions

The HPLC system (Shimadzu Corporation, Kyoto, Japan) in use with UV detection was a Shimadzu apparatus composed of LC-20AD pumps, DGU-20A5 degasser, SIL-20A auto sampler, CTO-20AC column thermostat connected with Valco column switching valve and SPD-M20A photodiode array (PDA) detector. The system coupled with mass detection was an Agilent 6120 quadrupole LC‑MS unit (Agilent Technologies, Santa Clara, California, USA). It was equipped with a quaternary pump, an inbuilt degasser, a column oven, an auto-sampler, a diode array detector, a single quadrupole MS with multi-mode source and a HP Chemstation software (Rev B.03.04 [16], Agilent Technologies, Santa Clara, California, USA).

The columns in use were CHIRALPAK ZWIX(+) and ZWIX(−) (3 µm particle size,150 × 3.0 mm and 250 × 4.0 mm i.d.) commercialized by Daicel Corporation (Tokyo, Japan) and manufactured at Chiral Technologies Europe (Illkirch, France). Other polysaccharide and crown ether-derived columns were used in the preliminary investigation, namely CHIRALPAK IE (5 µm particle size, 250 × 4.6 mm i.d.) and CROWNPAK CR-I(+) (5 µm particle size, 150 × 3.0 mm i.d.) commercialized by Daicel Corporation (Tokyo, Japan).

In the UV-compatible method, the optimized mobile phase used on CHIRALPAK ZWIX(+) (250 × 4.0 mm i.d.; 3 µm) was composed of 5 mM ammonium dihydrogen orthophosphate in MeOH/water (90/10, *v*/*v*). It was filtered through 0.45 µm membrane filter before use. The flow rate was set at 0.5 mL/min, column temperature at 10 °C, the injection volume at 30 µL, run time at 40 min and UV at 212 nm. The samples were dissolved in water/methanol (50/50, *v*/*v*) at a concentration of 10 mg/mL for injection.

In the MS-compatible method, the optimized mobile phase used on CHIRALPAK ZWIX(+) (150 × 3.0 mm i.d.,3 µm) was composed of 5 mM ammonium formate + 5 mM formic acid in MeOH/water (96/4, *v*/*v*). The flow rate was set at 0.5 mL/min, column temperature at 25 °C, the injection volume at 10 µL and run time at 10 min. MS detection was achieved on positive-ion mode using multimode electrospray interface at 200 °C with capillary voltage 1500 V and fragmentation voltage 70 V. The nebulizer and drying gases were nitrogen. Nebulizer pressure, drying gas flow and drying gas temperature were maintained at 50 psi, 10 L/min and 250 °C, respectively. Detection of the ions was performed in the single ion monitoring (SIM) mode at the mass to charge ratio *m*/*z* 160. The PRG racemic samples were dissolved to obtain a concentration of 1 ng/mL, 5 ng/mL and 15 ng/mL. The PRG API sample was prepared to obtain a test concentration of 10 µg/mL.

## 4. Conclusions

Two chromatographic methods were developed and optimized on columns packed with CSPs based on zwitterionic selectors, allowing the resolution of PRG enantiomers and the determination of chiral impurity with good sensitivities and with no need for derivatization. The optimization of the key parameters of the method based on the compatibility of the analysis with different detection modes ensured greatly enhanced detection performance. The limits of quantification (LOQs) were determined at 6 µg/mL in UV detection and 5 ng/mL by mass spectrometry. This latter value represents a 10‑fold improvement compared to the previously described method [12]. The method validation under the reported LC-MS conditions would be the objective of our further studies.

## Figures and Tables

**Table 1:** Effects of additive concentration, column temperature and water percentage on chromatographic parameters.

Investigated Parameters		Retention Times (min)		Resolution Degree (R_s_)	S/N Ratio	
		t_1_	t_2_	R_s_	(S/N)_1_	(S/N)_2_
	(mM)					
^(a)^ Effect of additive concentration	25	4.76	5.68	3.60	9.8	8.6
	20	4.93	5.97	3.98	7.5	7.5
	15	5.22	6.39	4.01	17.8	18.0
	10	5.24	6.32	4.04	15.0	13.7
	5	5.42	6.68	4.60	20.2	19.8
	(°C)					
^(b)^ Effect of temperature	15	6.32	8.04	4.62	32.0	28.5
	20	6.31	7.86	4.68	26.4	26.1
	25	6.20	7.61	4.62	24.3	22.5
	30	6.18	7.53	4.50	20.4	17.1
	35	6.05	7.39	4.45	18.3	15.7
	40	5.92	7.22	4.32	11.0	10.5
	(%)					
^(c)^ Effect of % of H_2_O	2	6.20	7.8	4.47	13.7	12.0
	3	5.73	7.04	4.19	23.7	21.1
	4	5.29	6.47	4.18	35.8	32.0
	5	4.97	6.07	3.93	24.3	22.4

^(a)^ MeOH/H_2_O (98/2, *v*/*v*) containing equal molar concentration of formic acid and ammonium formate, temperature: 25 °C, flow rate: 0.5 mL/min; ^(b)^ MeOH/H_2_O (98/2, *v*/*v*) containing 5 mM additive each, flow rate: 0.5 mL/min; ^(c)^ MeOH containing 5 mM additive each, temperature: 25 °C, flow rate: 0.5 mL/min.

**Table 2:** Resolution and S/N ratio values of (*R*)-enantiomer for different sample concentrations and injection volumes (API spiked with 0.15% of (*R*)-enantiomer).

Conc. of API (mg/mL)	Injection Volume (µL)	Sample Load (mg)	Resolution Degree (R_s_)	S/N Ratio of (*R*)-Enantiomer
3.0	100	0.30	1.45	9.0
5.0	30	0.15	2.44	8.2
5.0	60	0.30	1.56	9.0
8.0	20	0.16	2.24	5.8
8.0	40	0.32	1.45	11.3
10.0	10	0.10	2.90	7.5
10.0	20	0.20	1.98	15.0
10.0	30	0.30	1.54	21.0
10.0	40	0.40	1.49	21.0
10.0	50	0.50	1.26	26.0
10.0	60	0.60	1.07	46.0
15.0	20	0.30	1.61	11.7
